# Dual Kit/Aur Inhibitors as Chemosensitizing Agents for the Treatment of Melanoma: Design, Synthesis, Docking Studies and Functional Investigation

**DOI:** 10.1038/s41598-019-46287-5

**Published:** 2019-07-09

**Authors:** Luca Quattrini, Vito Coviello, Stefania Sartini, Teresa Di Desidero, Paola Orlandi, Yi-Yu Ke, Kai-Lun Liu, Hsing-Pang Hsieh, Guido Bocci, Concettina La Motta

**Affiliations:** 1 Dipartimento di Farmacia, Università di Pisa, Via Bonanno 6, 56126 Pisa, Italy; 2 Dipartimento di Medicina Clinica e Sperimentale, Università di Pisa, Via Roma 55, 56126 Pisa, Italy; 3 Institute of Biotechnology and Pharmaceutical Research, National Health Research Institutes, 35, Keyan Road, Zhunan Town, Miaoli County, 350 Taiwan

**Keywords:** Medicinal chemistry, Melanoma, Medicinal chemistry, Melanoma

## Abstract

Melanoma is the most serious form of skin cancer but its medication is still far from being safe and thoroughly effective. The search of novel therapeutic approaches represents therefore a health emergency to push through eagerly. In this study, we describe a novel class of dual c-Kit/Aur inhibitors, characterized by a 1,2,4-triazole core and developed by a structure-based optimization of a previously developed hit, and report the evidence of their significance as drug candidates for the treatment of melanoma. Compound **6a**, merging the best inhibitory profile against the target kinases, showed anti-proliferative efficacy against the human melanoma cell lines A2058, expressing the BRAF V600D mutation, and WM266-4, expressing BRAF V600E. Significantly, it displayed also a highly synergistic profile when tested in combination with vemurafenib, thus proving its efficacy not only *per se* but even in a combination therapy, which is nowadays acknowledged as the cornerstone approach of the forthcoming tumour management.

## Introduction

Melanoma is the most serious form of skin cancer, as it quickly metastasizes and poorly responds to conventional oncology treatments such as chemotherapy and radiation therapy^1^. Although accounting for a small percentage of all skin malignancies, its incidence and mortality rate are increasing faster than that of any other type of cancer. According to the American Cancer Society forecasts^2^, in 2018 there will be more than 91.000 novel cases of melanoma diagnosed in USA, with 2 to 3 cases occurring in men and more than 9.000 deaths in both sexes, and similar trends will be observed in both European and Eastern world^3^.

While early stage melanomas may be handled successfully with surgical resection, advanced and metastatic malignant diseases are inevitably characterized by poor prognosis and still represent an important health concern^4^. They have been treated for decades with immunotherapeutic agents like interferon alfa, cytokines and monoclonal antibodies, but both the marginal efficacy and the significant toxicity of these compounds have generally limited the median survival rate of the treated patients to six months^5^. Recently, an in-depth understanding of cell signalling pathways underlying melanoma initiation and progression opened up novel opportunities for targeted therapies, thus allowing to add innovative compounds to the pharmacological armamentarium for the treatment of this disease^6,7^.

Up to four main types of melanoma may be described, differentiated by anatomic sites and clinical features, as well as by distinctive genomic alterations including amplification, deletion and mutation of selected genes encoding for particular protein kinases. Actually, most of the more common melanomas arising from non-chronic sun-damaged skin are characterized by mutations in the gene encoding the serine-threonine kinase BRAF, the most frequent one being V600E, which stimulate tumour proliferation and survival *via* constitutive ERK signalling mediated by MEK^8^. Moreover, up to 30% of the same malignancies show gain-of-function mutation in the NRAS gene^9^, while PI3K mutations occur in up to 3% of metastatic melanomas^10^. On the contrary, melanomas affecting mucosal, acral and sun-damaged skin are characterized by mutations in the c-Kit gene, mainly at the 11, 13, 17 and 18 exons, or amplification of the c-Kit gene copy number. Eventually co-existing with oncogenic mutations affecting BRAF and NRAS^11^, c-Kit aberrations confer a constitutive tyrosine phosphorylation of the encoded receptor and a downstream activation of both the MAPK and the PI3K signalling cascade^12,13^.

Protein kinases represent therefore a sound target for the molecular therapy of people affected by melanomas and their inhibition is now pursued as an effective and viable therapeutic strategy^14^. Since 2011, four novel kinase inhibitors have been approved for the treatment of metastatic melanomas and malignancies that cannot be surgically removed. These include the pyrrolopyridine derivative vemurafenib^15^ (**1**, Chart 1-SI, Supplementary Information) and the thiazolylpyrimidine derivative dabrafenib^16^ (**2**, Chart 1-SI, Supplementary Information), which are able to block the V600 mutated BRAF protein, and the pyridopyrimidinetrione derivative trametinib^17^ (**3**, Chart 1-SI, Supplemental Information) and the azetidinmetanone derivative cobimetinib^18^ (**4**, Chart 1-SI, Supplemental Information), developed to inhibit the BRAF downstream kinase MEK. Although inducing a rapid and widespread response, which actually revolutionized the prognosis of patients increasing their median overall survival to two years or more, the use of these compounds is not without controversy and serious concern. In particular, their administration is limited to patients carrying the V600 BRAF mutation, thus malignancies characterized by a deregulation of different proteins, like c-Kit, still cannot benefit from a target therapy. Moreover, their use is accompanied by significant side effects, which are often challenging to manage and force to treatment interruption. In particular, the paradoxical activation of the MAPK pathway resulting from BRAF and MEK inhibition may accelerate pre-existing cancerous lesions^19,20^. Most importantly, these compounds trigger resistance mechanisms in the tumour cells, due to either re-activation of the targeted pathway or *de novo* activation of alternative signalling routes, thus making patients no longer responsive to both the initial therapy and additional treatment options^21–23^.

Therefore, even if the targeted therapy has definitively entered the clinical practice for the treatment of malignant melanoma, there is still a need to identify safe and thoroughly effective drugs. A step forward could be represented by the development of suitably tailored multi-effective kinase inhibitors, able to target at once key deregulated proteins affecting melanoma growth and survival. Besides increasing the therapeutic efficacy, these compounds should help, in principle, to reduce the emergence of resistance, above all when administered in combination with additional kinase inhibitors in customized cocktails of drugs^24,25^.

Among the different heterocyclic scaffolds exploited to build up novel anti-tumour protein kinase inhibitors, the 1*H*-1,2,4-triazole ring is getting more and more popular among medicinal chemists, who exploit this core increasingly for the design of novel compounds targeting the ATP-binding site of the proteins. Indeed, thanks to the 1,2-di-nitrogen substitution pattern, possibly assisted by the presence of further heteroatoms bound to positions 3 and 5, this nucleus is able to hook the kinase hinge region through H-bond interactions, thus allowing to obtain effective inhibitors. It is no coincidence that receptor-based virtual screening campaigns identify increasingly novel hits bearing the 1*H*-1,2,4-triazole ring as the main core, even when the studies are pursued against different kinases^26–28^. Actually, given the highly conserved structure of the hinge region across the wide kinase superfamily, this structural residue proves to bind both tyrosine and serine/threonine kinases, thus turning out to be highly handy for the development of multi-target inhibitors. A clear example of the drug design versatility of this nucleus is represented by compound DP01920, 5-(4-chlorophenyl)-3-((4-chlorophenylthio)methyl)-1*H*-1,2,4-triazole (**5**, Chart 1-SI, Supplemental Information), found out through a receptor-based virtual screening campaign aimed at obtaining novel inhibitors of RET, a tyrosine kinase receptor whose gain of function is causally linked to the development of different types of thyroid cancer^28^.

**Figure 1:**
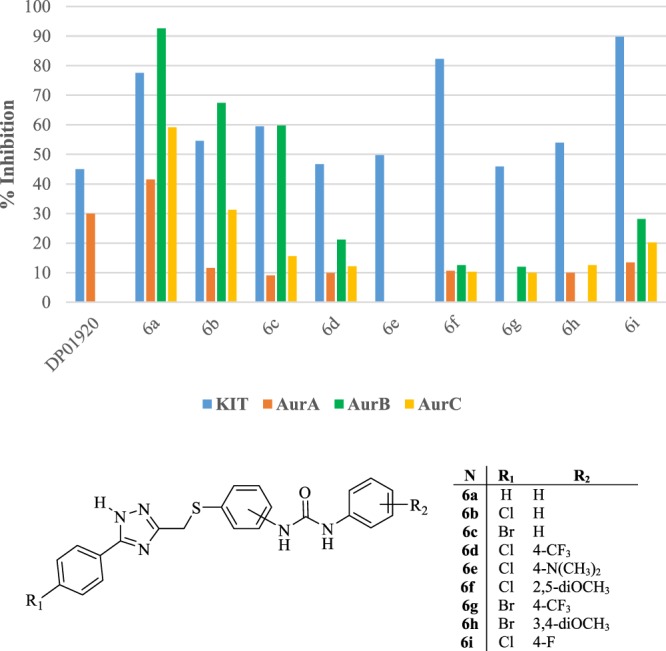
Kinase Inhibitory Data of 1-(Substituted)phenyl-3-(4-(((5-phenyl-1*H*-1,2,4-triazol-3-yl)methyl)thio)phenyl)urea Derivatives, **6a–i**. Data are expressed as percentage of kinase inhibition at 10 μM test compound, obtained as mean of at least three determinations. Standard error of the mean (SEM) is ≤10%. Compound **6e** displayed no inhibitory activity when tested against both AurA and AurB. Compound **6g** displayed no inhibitory activity when tested against AurA. Compound **6h** displayed no inhibitory activity when tested against AurB. The reference compound, DP01920, was not tested against both AurB and AurC.

Investigated against a panel of tyrosine and serine/threonine kinases, DP01920 revealed a multi-effective profile, proving to inhibit also the tyrosine kinase receptor c-Kit, which plays a key role in melanoma initiation and progression, and the serine/threonine kinase AurA, which is a master of cell division in coordination with the parent AurB and AurC (Fig. 1). Although displayed at concentrations in the 50–100 micromolar ranges, these ancillary activities made DP01920 the ideal hit compound to develop for the obtainment of multi-effective drug candidates for the treatment of melanoma. Accordingly, exploiting preliminary docking studies against the target kinases, we rationally optimized the 1,2,4-triazole derivative to obtain novel and effective dual c-Kit/Aur inhibitors.

Here we present a series of 1,2,4-triazole derivatives resulting from DP01920, whose aryl fragment in position 5 of the nucleus was suitably expanded through the insertion of phenyl-urea residues. The functional efficacy of the novel compounds were tested against the target proteins and melanoma cell lines, to prove the soundness of our optimization hypothesis. Moreover, seeking for a chemosensitizing agent to exploit for a combination therapy, which should enhance the efficacy of a standard drug reducing its side effects, the synergistic activity of the most effective 1,2,4-triazole derivative with the commercial agent vemurafenib was investigated as well, on melanoma cell lines proliferation.

## Results and Discussion

### Structural optimization of DP01920

**Figure 2:**
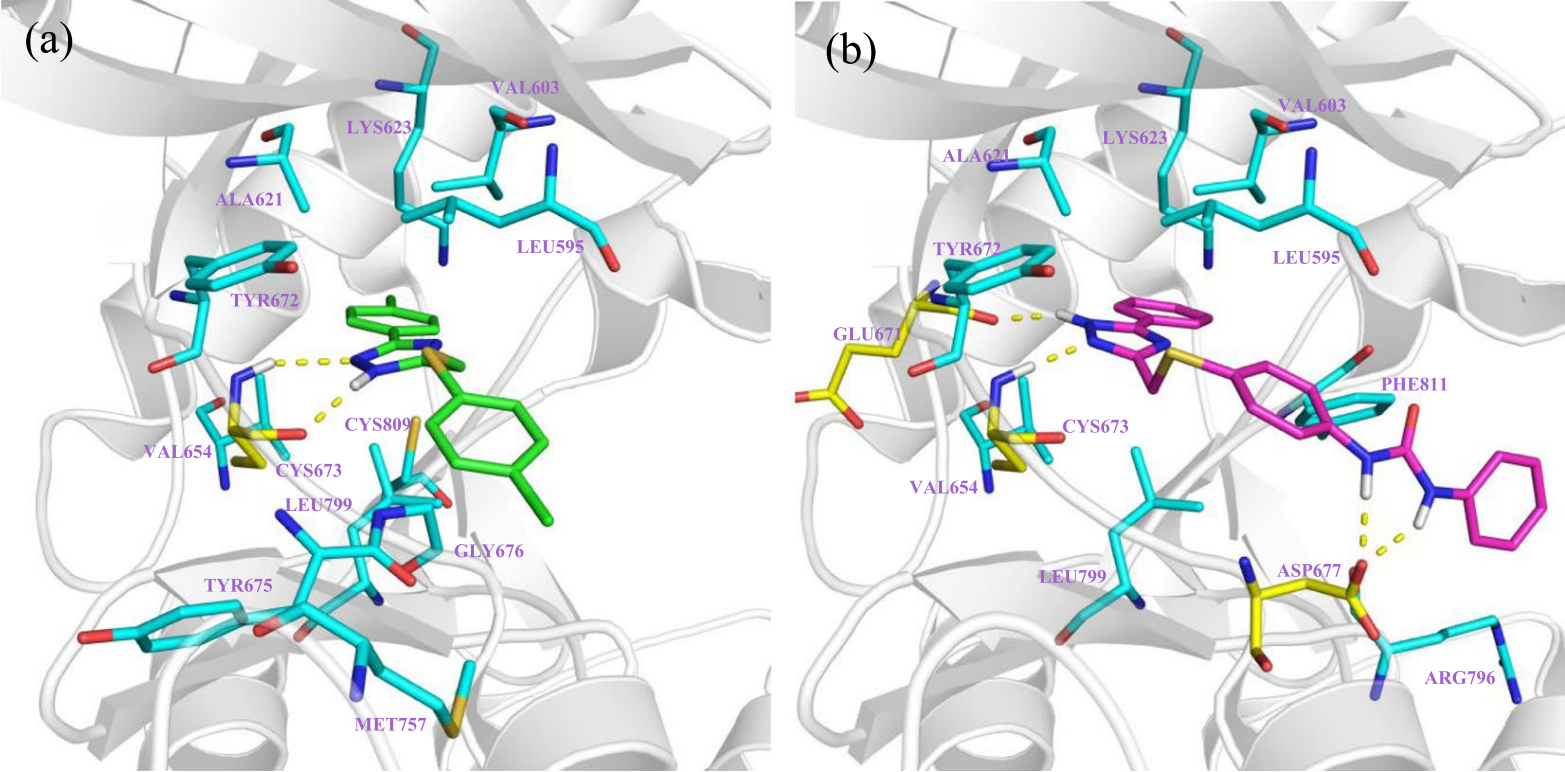
Binding pose of DP01920 (1a) and 1,2,4-triazole derivative **6a** (1b) into the active site of c-Kit. The protein is represented as grey ribbons and light blue sticks, H-bonds are represented as yellow dashed lines.

**Figure 3:**
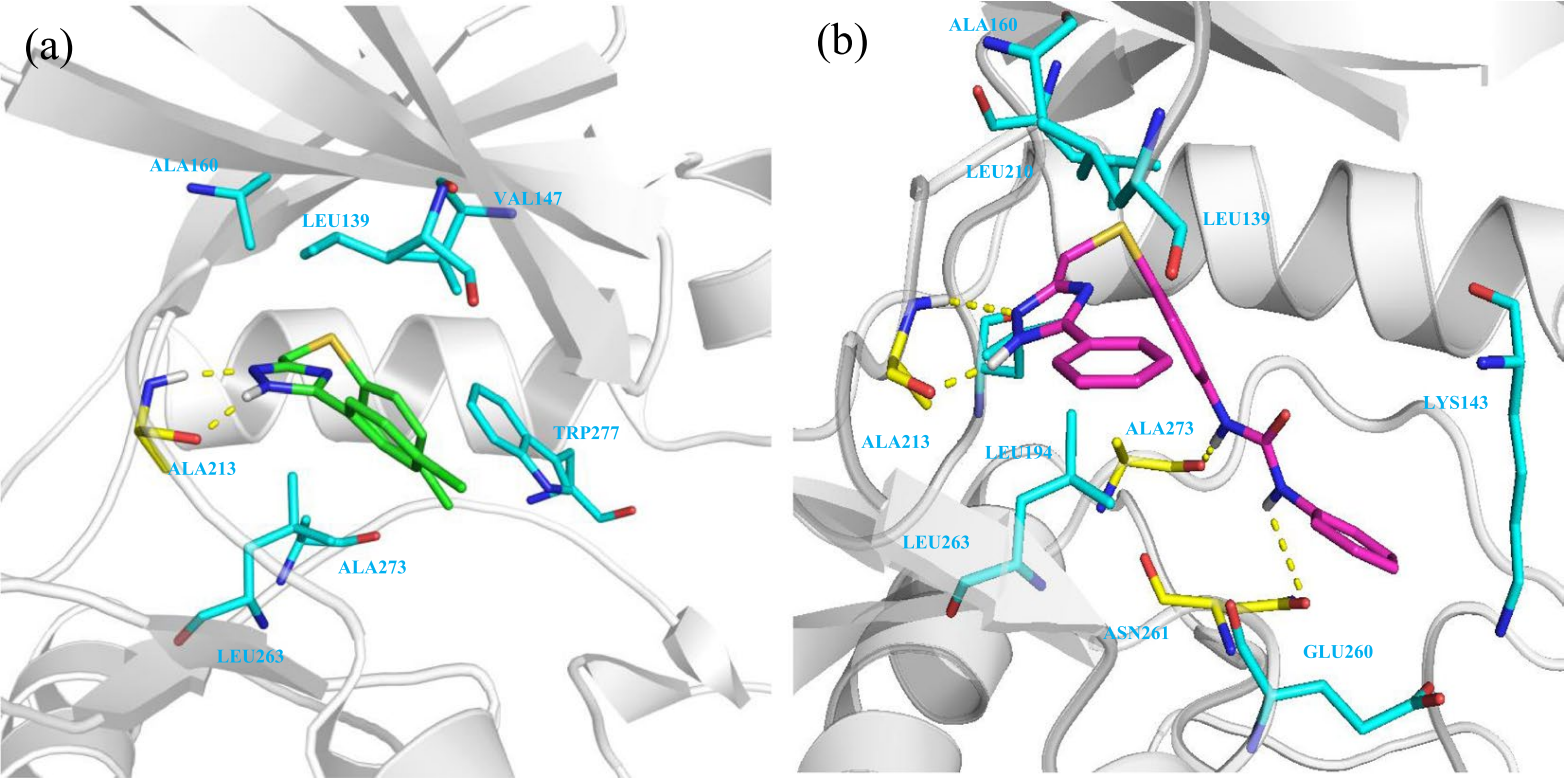
Binding pose of DP01920 (2a) and 1,2,4-triazole derivative **6a** (2b) into the active site of AurA. The protein is represented as grey ribbons and light blue sticks, H-bonds are represented as yellow dashed lines.

The rational optimization campaign of DP01920 started with an in-depth docking investigation of the compound into the ATP binding sites of the target protein kinases. Regarding c-Kit, Discovery Studio 2016//LigandFit program^29^ lodged DP01920 into the X-ray structure of the protein (PDB code: 3G0E^30^) so that the 1,2-di-nitrogen fragment of the triazole core can form a strong hinge binding with Cys673. Moreover, the 5-pheny fragment fits into the deep end of the site setting additional hydrophobic interactions with Leu595, Val603, Ala621, Lys623, Val654, Tyr672, Tyr675, Gly676, Met757, Leu799, and Cys809, thus strengthening the binding of DP01920 with the backbone of the protein (Fig. 2a). Similar outcomes were obtained docking the compound into the X-ray structure of AurA (PDB code: 5EW9^31^). Indeed, also in this case, the 1,2-di-nitrogen fragment of the core turned out to be a key residue, as it gears the molecule into the ATP site of the protein H-bonding Ala213 (Fig. 3a). In doing so, it casts the 5-phenyl ring toward the aromatic Trp277, thus allowing a pi-pi stacking interaction which makes the DP01920 linking with the AurA binding site more effective. The obtained binding poses into the active sites of both c-Kit and AurA offer interesting clues for the rational optimization of the hit. In particular, they lead to speculate that the insertion of suitable substituents on the pendant phenyl rings may help to fill in further and more specifically the binding pockets of the two proteins, which turn out to be only partially occupied by the relatively small DP01920. To this end, the insertion of the aryl-urea fragment, often exploited for the development of multi-kinase inhibitors like sorafenib^32^, regorafenib^33^, AT-9283^34^ and many others, could come in very handy. Actually, it merges an aromatic portion, able to face the lipophilic aminoacid residues surrounding the site, with nitrogen and oxygen atoms, which can take part in H-bonds with the backbone of the proteins both as donor and acceptor fragments. Thus, inserting this structural residue on the 5-phenyl-thiometyl portion of the DP01920 hit we obtained the 1-phenyl-3-(4-(((3-phenyl-1*H*-1,2,4-triazol-5-yl)methyl)thio)phenyl)urea derivative **6a** (Chart 1-SI; Supplemental Information) as the representative compound of a novel dual c-Kit/Aur inhibitor. A preliminary docking study of **6a** into the ATP binding site of c-Kit was then undertaken, to verify the soundness of our design hypothesis. As clearly showed in Fig. 2b, the 1,2-N(H)-N fragment of the core is still engaged in hydrogen bonding with the backbone, hooking Glu671 and Cys673 residues, while the extended urea group, chosen to optimize the DP01920 hit, forms a strong hydrogen bonding with Asp677. Therefore, this residue plays in principle a key role in bonding the active site of the protein suggesting that the compound has the potential to inhibit efficiently the target kinase. Similar results were also obtained docking the **6a** lead into the active site of AurA (Fig. 3b). The heterocyclic core of the compound maintains a fruitful H-bond interaction with Ala213, as in the case of DP01920, while the added aryl-ureic fragment H-bonds both Ala273 and Asn261, thus proving to be actively involved in the interaction with the protein. The physicochemical properties and the druglikeness profile of **6a**, including PAINS estimation, were then evaluated in silico, exploiting the SwissADME facility^35^, and the obtained promising results (Table 1-SI, Supplemental Information) finally allowed to account this compound as a novel lead to invest in. Different substituents were then placed on the distal phenyl ring of the aryl-ureic fragment, showing either an electron withdrawing or an electron donating profile, in order to investigate the structure-activity relationships of these novel class of dual-kinase inhibitors.

### Synthesis of novel 1,2,4-Triazole derivatives

The synthesis of the target inhibitors **6a–i** was performed as depicted in Fig. 1-SI (Supplemental Information). Reaction of either 4- or 3-aminobenzenethiol **7a**,**b** with ethyl chloroacetate and K_2_CO_2_ gave the corresponding ethyl esters **8a**,**b**, which were converted into acetohydrazides **9a**,**b** by treatment with hydrazine in methanol solution. Cyclization of **9a**,**b** to the 1,2,4-triazole derivatives **10a–d** was accomplished by reaction with the suitable benzonitrile, in the presence of K_2_CO_3_. The key 1,2,4-triazole intermediates afforded the desired ureic inhibitors **6a-i** by reaction with substituted phenylisocyanates in THF solution.

### Functional evaluation of novel 1,2,4-Triazole derivatives

The novel synthesized compounds were tested for their inhibitory properties against the target proteins, c-Kit and AurA, as well as the parent mitotic AurB and C, as they all have control over cell proliferation. As reported in Fig. 1, listing functional activities expressed as percentage inhibition at 10 μM, all the novel compounds showed a remarkable enhancement in inhibitory efficacy against the main targets, c-Kit and AurA, when compared to the reference hit, DP01920, thus corroborating the functional significance of the aryl-urea fragment proposed by our design hypothesis. Regarding the tyrosine kinase c-Kit, the activity of the novel unsubstituted aryl-ureic lead **6a** was increased by the concurrent insertion of an electron-withdrawing chloro atom on the 5-phenyl ring and a 2,5-dimethoxy substitution pattern on the distal phenyl ring, as in the case of **6f**. On the contrary, the presence of any other different combination of both electron-donating and electron-withdrawing atoms on the core gave rise to a slight reduction of its inhibitory potency. Significantly, moving the aryl-ureic fragment on the 5-phenyl-thiomethyl portion from position para to position meta, thus bending the geometry of the molecular skeleton, gave rise to an excellent inhibitory profile and derivative **6i**, characterized by a 4-fluoro substituent on the distal phenyl ring, turned out to be the most effective compound of the whole series. In the case of AurA, the lead **6a** proved to be the most active of the synthesized series, and major results were also obtained against the parent AurB. The compound gets settled nicely into the kinase domain of the two proteins but, at the same time, it leaves little room for structural modifications. Actually, the insertion of any type of substituent, regardless of its steric and electronic contribution, determined a slight to significant reduction of its inhibitory efficacy. A similar functional trend was observed testing the compounds against AurC. Indeed, also in this case, the unsubstituted lead, **6a**, tuned out to be the most effective of the whole series and the increase of structural complexity through the gradual addition of substituents at one end, as in **6b**,**c**, and at both ends of the molecule, as in **6d–i**, lead to a progressive decrease of activity.

The observed functional data were rationalised through molecular modelling analyses. Regarding c-Kit, docking studies of the most effective compounds, **6a**,**f**,**i**, resulted in pretty similar poses, as they are all kept into the active site of the protein thanks to the strong H-bond interactions with Glu671, Cys673, and Tyr672 (Fig. 2-SI, Supplementary Information).

**Figure 4:**
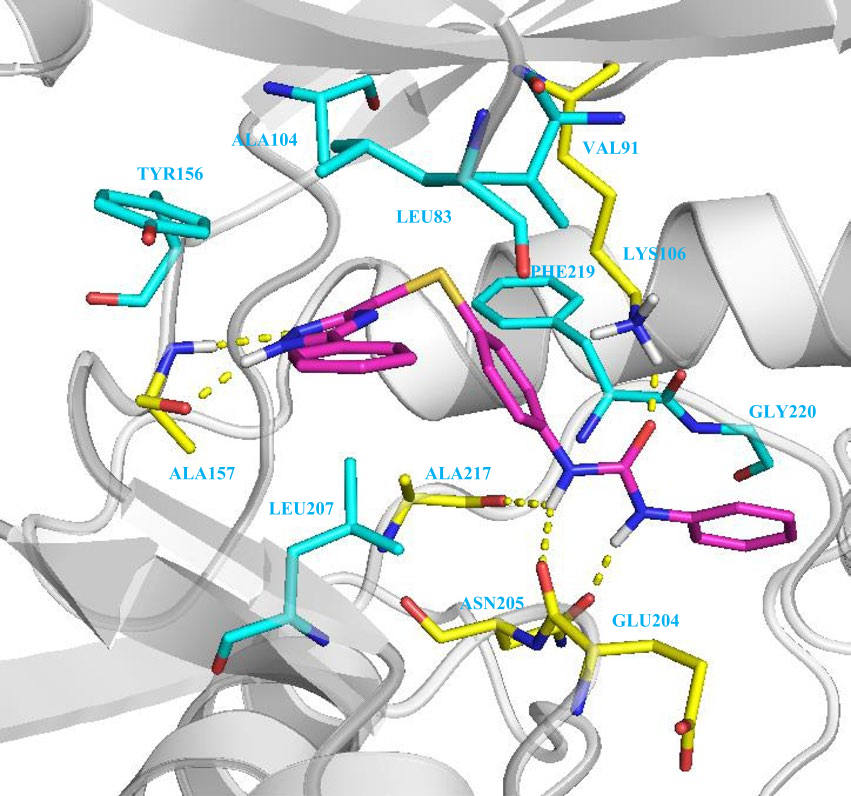
Binding pose of derivative **6a** into the active site of AurB. The protein is represented as grey ribbons and light blue sticks, H-bonds are represented as yellow dashed lines.

Regarding AurA, none of the structural changes made to the unsubstituted **6a** turned out to be satisfactory. Actually, the chosen substitutions induced steric clashes with the Aurora binding pocket influencing the compounds’ hinge binding, and this is why they all showed lower inhibitory efficacy against the protein. In the case of AurB, the docking study of compound **6a** into the X-ray structure of the protein (PDB code: 4AF3^36^) highlights the tight bond between the urea fragment and the Lys106, Glu204, Asn205, and Ala217 residues. Moreover, the flanking aromatic rings have strong hydrophobic interaction with Phe219 and Gly220 (Fig. 4), and the resulting network of contact, not found in the AurA binding pose (Fig. 3-SI, Supplemental Information), well justifies the almost double difference in inhibitory efficacy of the compound between the two protein isoforms.

**Figure 5:**
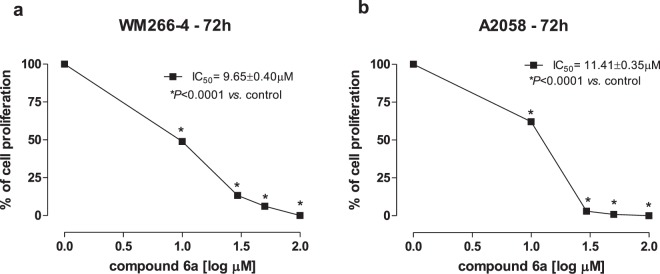
Antiproliferative activity of 1,2,4-Triazole **6a** on BRAF V600E WM-266-4 (5a) and BRAF V600D A-2058 (5b) cell lines.

Compound **6a**, merging the best inhibitory profile against the target c-Kit/AurB kinases, was tested on the human A2058 (American Type Culture Collection, Manassas, USA) and WM266-4 (European Collection of Authenticated Cell Cultures, Salisbury, UK) cell lines, chosen as representative of *BRAF-*mutated melanoma cell lines, to investigate its anti-proliferative efficacy. In particular, the WM266-4 cell line harbors the BRAF V600E mutation, whereas the A2058 cells has the BRAF V600D one. Vemurafenib, the BRAF V600E inhibitor currently used for the treatment of inoperable, metastatic melanoma, was used as the reference compound. After 72 hours of exposure, the test compound **6a** proved to block cells growth in a dose-dependent manner, showing comparable IC_50_ values for both the lines: 9.65 μM (Fig. 5a) and 11.41 μM (Fig. 5b) for WM266-4 and A2058, respectively. Moreover, as combination therapy with drugs given simultaneously is becoming by far the most effective approach to treat clinically challenging diseases, including melanoma^37^, **6a** was tested in combination with vemurafenib to investigate the efficacy of a combination strategy targeting malignancies characterized by a deregulation of different kinases. The level of possible interaction between the two kinase inhibitors, synergistic, additive or antagonist in nature, was determined by the method of Chou-Talaly^38^ and quantitated through the combination index (CI), calculated exploiting the following multiple drug-effect Eq. (1)

1 $${\rm{CI}}=[{({\rm{D}})}_{1}/{({\rm{Dx}})}_{1}]+[{({\rm{D}})}_{2}/{({\rm{Dx}})}_{2}]$$

and referred to as synergism, additive effect and antagonism when CI < 1, CI = 1 and CI > 1, respectively. As an example, at the 95% inhibition level, (Dx)_1_ and (Dx)_2_ are the concentrations of **6a** and vemurafenib, respectively, that induce a 95% inhibition of cell proliferation; (D)_1_ and (D)_2_ are the concentrations of compound **6a** and vemurafenib in combination that also inhibit cell proliferation by 95% (isoeffective as compared with the single drugs alone).

**Figure 6:**
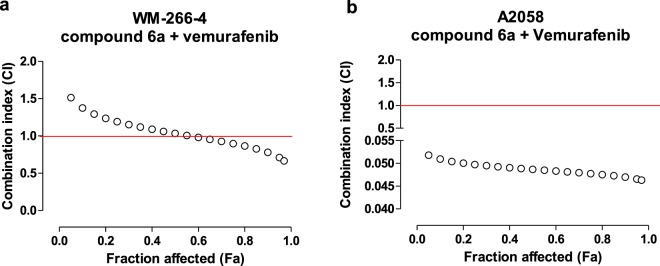
Synergistic activity of 1,2,4-Triazole **6a** and vemurafenib association on WM-266-4 (6a) and on A-2058 (6b) cell lines, expressed as combination index (CI) values. The red line represents the additive effect. CI < 1 values represent synergism, CI = 1 additive, and CI > 1 antagonistic activity between the two drugs.

As summarized in Table 1, the simultaneous exposure of WM-266-4 cells to different concentrations of **6a** and vemurafenib for 72 h showed a synergism for affected fraction (Fa) of cells higher than 50% (CI < 1, Fig. 6a), and, in particular, for affected fraction ranging from 75 to 95%. A highly synergistic profile was also found for simultaneous combination **6a** and vemurafenib for 72 h in A2058 cell line (CI < 1, Fig. 6b), for all the affected fraction of cells.

**Table 1:** Synergistic activity of **6a** and vemurafenib association expressed as both combination index (CI) and dose reduction index (DRI) values for each drug at 75%, 85% and 95% inhibition of A2058 and WM266-4 cell proliferation.

Affected Cell Fraction (%)	Combination Index (CI) 6a + vemurafenib		Dose Reduction Index (DRI) 6a + vemurafenib			
	A2058	WM266-4	A2058		WM266-4	
			6a	vemurafenib	6a	vemurafenib
75	0.048	0.897	494.1	21.8	374.9	1.1
85	0.047	0.827	429.0	22.2	272.1	1.2
95	0.046	0.710	327.9	22.9	147.9	1.4

The Dose Reduction Index (DRI) was calculated as well, to assess the theoretical magnitude of concentration reduction allowed for the two drugs when given in synergistic combination *in vitro* to achieve the same effect as that obtained with the concentration of each single agent. DRI was calculated by the following Eq. (2):

2 $$({\rm{DRI}})=({\rm{Dx}})/({\rm{D}})$$

Indeed, in the cases of **6a** or vemurafenib, it could be possible to reduce the concentration of the drug *in vitro* more than 300-fold or 20-fold (Table 1), respectively, when the drugs are combined to obtain the same 95% level of cytotoxic effects in A-2058 cells, whereas in WM266-4 cells the possible dose reduction is markedly lower for **6a** (i.e. 150-fold) and vemurafenib (1.4-fold). Of note, synergism and related reductions of drug concentrations are extremely important for high levels of cell proliferation inhibition (e.g. >75%) to obtain a clinical advantage such as the reduction of tumor mass or the control of neoplastic disease.

The decision to test the effects of the compound **6a** on WM266-4 and A-2058 cell lines was due to their different BRAF mutational status. Indeed, the antiproliferative activity of the **6a** was independent from the BRAF mutational status of the melanoma cell lines, as witnessed by the superimposable IC_50_s. Furthermore, the synergistic and chemosensitizing effect was obtained mainly in the BRAF V600D cell line, that responded less to the treatment with vemurafenib alone, thus suggesting the possible use of this combination in future clinical studies enrolling patients with this rare mutation but less therapeutic options. However, the **6a** effects should be tested on other BRAF V600E melanoma cell lines to confirm the cell proliferation inhibition and the synergism with the target therapy vemurafenib.

**Figure 7:**
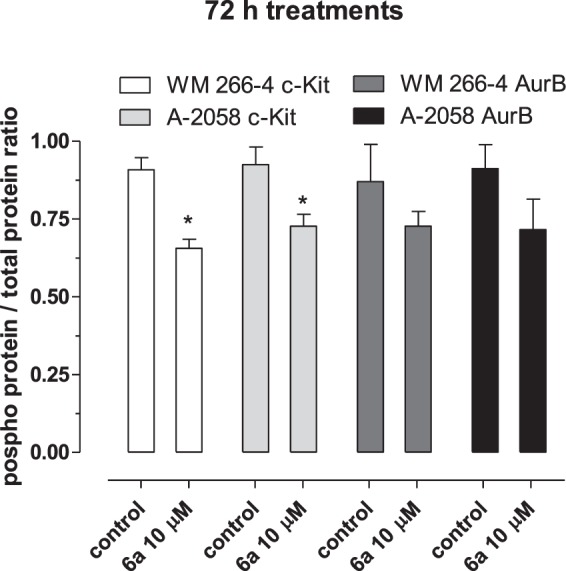
Inhibition of c-Kit and AurB phosphorylation by compound **6a** in WM-266-4 and A-2058 cells after 72 h of treatment. The data are expressed as the ratio of phosphorylated c-Kit or AurB/total c-Kit or AurB. Columns and bars, mean values ± SD, respectively. *P < 0.05 vs. vehicle-treated controls.

Interestingly, compound **6a** showed also a significant inhibition of c-Kit phosphorylation on both cell lines at concentrations that significantly inhibited ≥50% of proliferation of melanoma cells, as outlined by Fig. 7. After exposure to **6a** concentrations, the quantity of the phosphorylated form of AurB in both cell lines (Fig. 7) was weakly reduced after 72 h, but not reached a statistical significance if compared to the vehicle-treated samples.

## Concluding Remarks

In this work we described a novel class of 1,2,4-triazole derivatives possessing a peculiar dual c-Kit/AurB inhibitory profile. Obtained from a previously described hit^28^ by means of a structural improvement, pursued through a rational approach facilitated by preliminary docking studies, the novel compounds are able to target two key protein kinases accountable for both pathogenesis and progression of melanoma. Actually, it is a fact that malignancies arising particularly from acral, mucosal, and chronically sun-damaged sites harbor c-Kit alterations^11–13^, and it has recently become clear that both expression and activity of the serine-threonine AurB is remarkably increased during both BRAF wild type and V600 mutated melanoma progression^39,40^.

Among the synthesized compounds, derivative **6a**, 1-phenyl-3-(4-(((3-phenyl-1*H*-1,2,4-triazol-5-yl)methyl)thio)phenyl)urea, emerged as a promising lead. Besides showing the most efficient combination of inhibitory activity against the target kinases, **6a** displayed anti-proliferative efficacy when tested against human A2058 and WM266-4 melanoma cell lines. Significantly, it also exhibited highly synergistic properties when administered to the same cell lines as a simultaneous combination with the known inhibitor vemurafenib. Therefore, **6a** represents an original prototype of kinase inhibitor whose functional profile may provide, in principle, a novel and viable chance to treat melanoma by means of a targeted and sinergistic approach. Clearly, thorough investigations in animal models of melanoma are now necessary, to both prove the effectiveness of **6a** as a chemosensitizing agent and verify the safe of the proposed combination therapy, as the concomitant administration of different targeted drugs may be not free from adverse side effects.

## Methods

### Chemistry

Melting points were determined using a Reichert Köfler hot-stage apparatus and are uncorrected. Routine ^1^H-NMR and ^13^C spectra were recorded in DMSO-d_6_ on a Bruker 400 spectrometer operating at 400 MHz. Evaporation was performed in vacuo (rotary evaporator). Analytical TLC analyses were carried out on Merck 0.2 mm precoated silica gel aluminium sheets (60 F-254). Purity of the target inhibitors, **6a–g**, was determined by HPLC analysis, using a Merck Hitachi D-7000 liquid chromatograph (UV detection at 242 nm) and a Discovery C18 column (250 mm × 4.6 mm, 5 μm, Supelco), with a gradient of 40% water and 60% methanol and a flow rate of 1.4 mL/min. All the compounds showed percent purity values ≥95%. 4-Aminobenzenethiol, 3-aminobenzenethiol, benzonitrile, 4-chlorobenzonitrile, 4-bromobenzonitrile and all the suitably substituted phenylisocyanates, used to obtain the target inhibitors, were from Sigma-Aldrich and Fluka.

### Synthesis of Ethyl 2-((4-aminophenyl)thio)acetate and Ethyl 2-((3-aminophenyl)thio)acetate, 8a,b

A solution of the suitable aminobenzenethiol (1.00 mmol), chloroethylacetate (1.00 mmol), and K_2_CO_3_ in DMF was left under stirring at room temperature until the disappearance of the starting material (TLC analysis). Once reaction was complete, the solvent was removed in vacuo and the resulting residue was diluted with water and extracted with running portions of AcOEt. The combined organic portions were then dried over sodium sulfate and the solvent removed in vacuo. The crude product was purified by flash chromatography (eluting mixture AcOEt/Hexane 3/7), and characterized through physical and spectral data (Table 2-SI, Supplemental Information).

### Synthesis of 2-((4-Aminophenyl)thio)acetohydrazide and 2-((3-Aminophenyl)thio)acetohydrazide, 9a,b

The ester derivative **8a**,**b** (1.00 mmol) was allowed to react with hydrazine (3.00 mmol) in EtOH solution, at T = 100 °C, until the disappearance of the starting material (TLC analysis). The solvent was then removed in vacuo and the solid obtained was purified by recrystallization from EtOH, then characterized through physical and spectral data (Table 2-SI, Supplemental Information).

### General procedure for the synthesis of 3- and 4-(((5-(Substituted-phenyl)-1*H*-1,2,4-triazol-3-yl)methyl)thio)aniline, 10a–d

A mixture of the thioacetohydrazide **9a**,**b** (1.00 mmol), the suitably substituted nitrile (3.00 mmol), and K_2_CO_3_ (0.5 mmol) in *n*-BuOH (2 mL) was heated under stirring at 150 °C in a sealed vial until the disappearance of the starting material (TLC analysis). The solvent was then removed in vacuo and the residue obtained was diluted with water and extracted with AcOEt. The combined organic extracts were dried over sodium sulfate and the solvent removed in vacuo. The crude product was finally purified by flash chromatography (eluting mixture AcOEt/Hexane 5/5), then recrystallized from suitable solvent and characterized through physical and spectral data (Table 2-SI, Supplemental Information).

### General Procedure for the Synthesis of 1-Phenyl-3-(3-(((5-phenyl-1*H*-1,2,4-triazol-3-yl)methyl)thio)phenyl)urea, 6a–g

A solution of the 1,2,4-triazole derivative **10a–d** (1.00 mmol) and the appropriate phenyl isocyanate (1.00 mmol) in THF was left under stirring at room temperature until the disappearance of the starting material (TLC analysis). Once the reaction was complete, the solvent was removed *in vacuo* and the residue obtained was purified by recrystallization from the suitable solvent and characterized through physical and spectral data (Table 3-SI, Supplemental Information).

## Biology

### Materials and Methods

Cell culture media EMEM and DMEM, fetal bovine serum (FBS), L-glutamine, and antibiotics were from Sigma-Aldrich St. Louis, MO, USA). Vemurafenib was purchased from Selleckchem (Munich, Germany). Human recombinant protein kinases and Protein kinase assay kits were from ThermoFisher Scientific (MA, US). Plastics were supplied by Sarstedt (Verona, Italy).

### Protein kinases inhibitory assay

Inhibitory assays were performed in accordance with a previously reported procedure, standardized for RET and VEGFR2^28,41^. Test compounds were dissolved in 100% DMSO and diluted to the appropriate concentrations with the reaction buffer, provided by the kit. The final concentration of DMSO in assay solutions never exceeded 1%, and proved to have no effects on protein activity. The inhibitory effect of the novel compounds was routinely estimated at 10 μM concentration.

### Proliferation assay

Test compounds **6a** and vemurafenib were dissolved in a stock solution of 10 mM in 100% dimethylsulfoxide (DMSO) for *in vitro* studies. DMSO concentration in the control’s media was the same used to make up the highest concentration of test compounds in growth media for the same experiment. *In vitro* chemosensitivity was tested on melanoma WM266-4 and A2058 cell lines. Cells were grown in EMEM and DMEM media, respectively, plated in sterile 24-well plastic plates and treated for 72 h (using 10^3^ cells/well in 1 mL of medium) with added **6a** (range of 1–100 μM), or vemurafenib (0.0001–100 μM) alone. The synergistic effect between **6a** and vemurafenib was calculated with the method of Chou-Talaly^38^ based on the multiple drug-effect equation and quantitated by the combination index (CI) and the dose reduction index (DRI), where CI < 1 and DRI > 1 indicate synergism. At the end of the experiment, cells were harvested with trypsin/EDTA, and viable cells were quantified using the automatic cell counter ADAM MC Digital B (Twin Helix, Milano, Italy). The data are presented as the percentage of vehicle-treated cells. The concentration of drugs that decreased cell count by 50% (IC_50_) compared with controls was calculated by nonlinear fitting of experimental data. All experiments were repeated independently three times with at least nine samples for each concentration.

### c-Kit and AurB ELISA assay

WM266-4 and A2058 cells (5 × 10^4^ cells/well) were treated for 72 h with compound **6a** (10 μM) or with vehicle alone. To measure phospho c-Kit and phospho AurB, at the end of the experiment, the cells were harvested and immediately frozen with liquid nitrogen. Cells were then lysed and the total protein was measured. In each sample, an equal amount of proteins was then assayed for human c-Kit and AurB phosporylation by the Human Phospho-CD117/c-kit ELISA Kit (Assaysolution; Woburn, MA, USA) and the AurB (Phospho-Thr232) ELISA Kit (Aviva System Biology; San Diego, CA, USA) and normalized by total protein c-Kit and AurB concentration measured by c-Kit and AurB ELISA kits, respectively. The optical density was determined using the microplate reader Multiskan Spectrum set to 450 nm. All experiments were repeated, independently, six times with at least nine samples for each concentration. The data were expressed as the ratio of phosphorylated c-Kit or AurB/total c-Kit or AurB.

### Molecular modeling study

The c-Kit (PDB code: 3G0E^30^), Aurora-A (PDB code: 5EW9^31^), and Aurora-B structures (PDB ID: 4AF3^36^) were used for the docking study. The docking analysis was conducted using the Discovery Studio 2017//LigandFit program with the CHARMm force field^42^. The number of docking poses was set as 100 with default parameters. The PDB models selection and validation, as well as the choice of the best conformations were determined as previously described, according to the complex binding structure obtained with the same proteins^43–46^.

## Supplementary information

Supplementary Info

## Data Availability

All data generated or analysed during this study are included in this published article (and its Supplementary Information Files).
