# Supplementary material for: Dual Kit/Aur Inhibitors as Chemosensitizing Agents for the Treatment of Melanoma: Design, Synthesis, Docking Studies and Functional Investigation
Source: Sci Rep. 2019 Jul 9;9:9943. doi: 10.1038/s41598-019-46287-5 (PMC6617451; doi:10.1038/s41598-019-46287-5)
Supplement: Supplementary file 1 — Supplementary Info [file 41598_2019_46287_MOESM1_ESM.pdf]

Supplemental Information

Dual Kit/Aur Inhibitors as Chemosensitizing Agents for the Treatment of Melanoma: Design, Synthesis, Docking Studies and Functional Investigation.

Luca Quattrini,[ς] Vito Coviello,[ς] Stefania Sartini,[ς] Teresa di Desidero,[§] Paola Orlandi,[§] Yi-Yu Ke,[£] Kai-Lun Liu,[£] Hsing-Pang Hsieh,[£] Guido Bocci,[§] Concettina La Motta.[ς*]

[ς]*Dipartimento di Farmacia, Università di Pisa, Via Bonanno 6, 56126 Pisa, Italy*

[§]*Dipartimento di Medicina Clinica e Sperimentale, Università di Pisa, Via Roma 55, 56126 Pisa, Italy.*

[£]*Institute of Biotechnology and Pharmaceutical Research, National Health Research Institutes, 35, Keyan Road, Zhunan Town, Miaoli County 350, Taiwan.*

Chart 1-SI

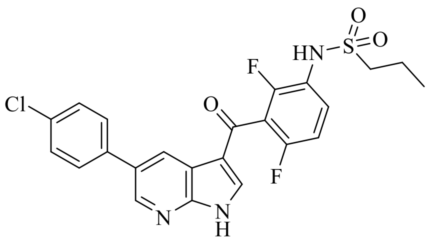

1, Vemurafenib

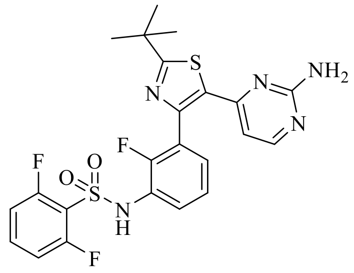

2, Dabrafenib

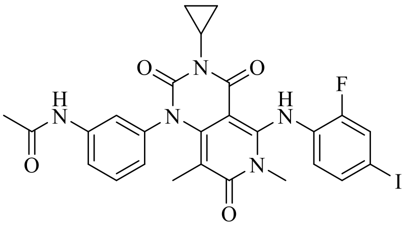

3, Trametinib

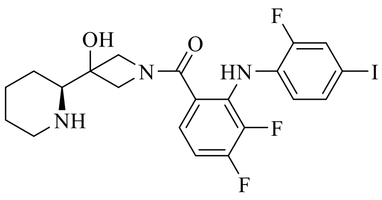

4, Cobimetinib

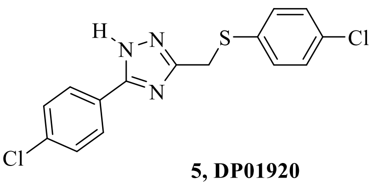

5, DP01920

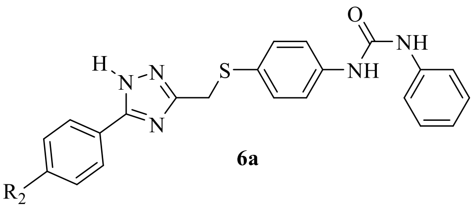

6a

Chart 1. Commercial and Investigational Kinase Inhibitors

Table 1-SI. Physiochemical Properties and Druglikeness Profile of Derivative **6a**.

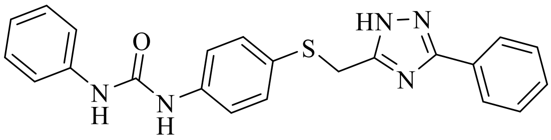

Physicochemical Properties	
Molecular weight	401.48 g/mol
Num. heavy atoms	29
Num. arom. heavy atoms	23
Fraction Csp3	0.05
Num. rotatable bonds	8
Num. H-bond acceptors	3
Num. H-bond donors	3
Molar Refractivity	116.75
TPSA	108.00 Å²
**Lipophilicity**	
Consensus Log $P_{o/w}$	3.75
**Water Solubility**	
Log *S* (ESOL)	-5.07
Solubility	3.45e-03 mg/ml ; 8.60e-06 mol/l
Class	Moderately soluble
**Pharmacokinetics**	
GI absorption	High
BBB permeant	No
P-gp substrate	No
**Druglikeness**	
Lipinski	Yes; 0 violation
Ghose	Yes
Veber	Yes
Egan	Yes
Muegge	Yes
Bioavailability Score	0.55
PAINS	0 alert
Brenk	0 alert

Figure 1-SI. Synthesis of 1-substitutedphenyl)-3-(4-(((5-phenyl-1H-1,2,4-triazolo-3-yl)methyl)thio)phenyl)urea, **6a-i**.

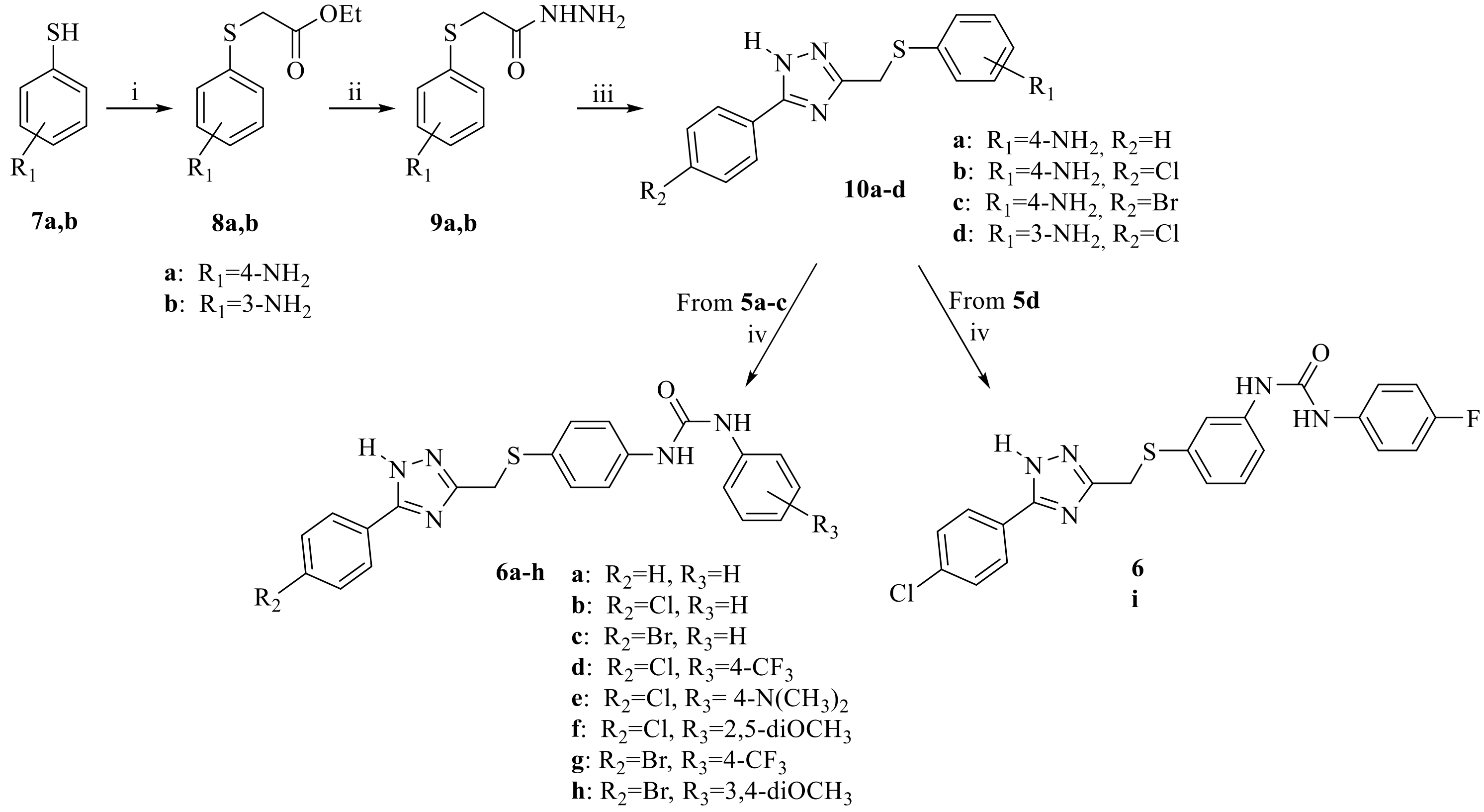

Reagents and conditions:
i) $ClCH_2COOEt$, K_2CO_3, DMF, r.t.; ii) NH_2NH_2, MeOH, Δ; iii) (4-Substituted)benzonitrile, K_2CO_3, BuOH, Δ; iv) Subtituted-phenyl isocyanate, THF, r.t.

Figure 2-SI. Binding pose of derivatives **6a** (magenta), **6f** (yellow), and **6i** (light blue) into the active site of c-Kit, shown as grey area.

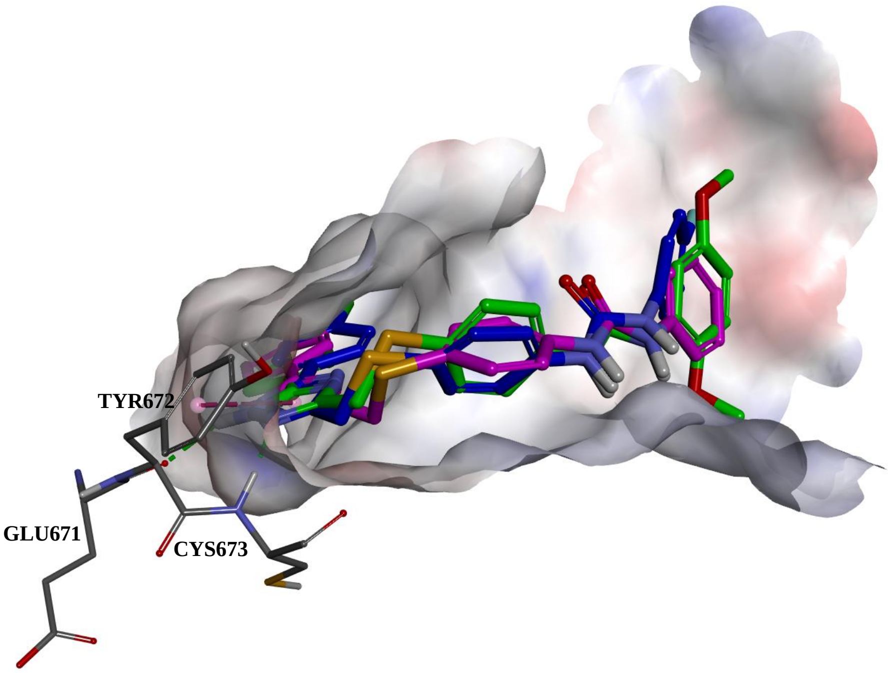

Figure 3-SI. Comparison between the 2D binding pose of derivative **6a** into the active sites of AurA (left) and AurB (right). Conventional hydrogen bonds are represented as green dashed lines, pi-pi and amide-pi stacking are represented as dark pink dashed lines, and pi-alkyl stacking are represented as light pink dashed lines.

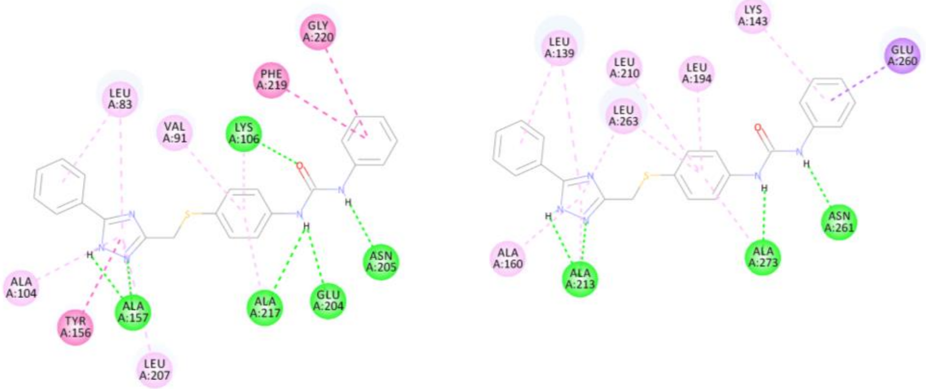

Table 2-SI. Physical and Spectral Data of Key Intermediates 8a,b, 9a,b, 10a,d.

**Ethyl 2-((4-aminophenyl)thio)acetate, 8a.** Yellowish oil. Yield: 79%. ^{1}H-NMR (δ, ppm): 7,12 (d, 2H, J=8,60 Hz), 6,51 (d, 2H, J=8,60 Hz), 5,33 (s, 2H, exc.), 4,03 (q, 2H, J=7,11 Hz), 3,48 (s, 2H), 1,13 (t, 3H, J=7,10 Hz).
**Ethyl 2-((3-aminophenyl)thio)acetate, 8b.** Yellowish oil. Yield: 75%. ^{1}H-NMR (δ, ppm): 7,09 (t, 1H, J=7,80 Hz), 6,54 (s, 1H), 6,46 (d, 1H, J=7,60 Hz), 6,41 (d, 1H, J=8,00 Hz), 4,09 (q, 2H, J=7.10 Hz), 3,74 (s, 2H), 1,16 (t, 3H, J=7.10 Hz).
**2-((4-Aminophenyl)thio)acetohydrazide, 9a.** White solid. M.p. 94-96 °C. Yield: 90%. ^{1}H-NMR (δ, ppm): 9.03 (s, 1H, exc.), 7.11 (d, 2H, J=8.56 Hz), 6.51 (d, 2H, J=8.57 Hz), 5.24 (s, 2H, exc.), 4.21 (s, 2H, exc.), 3.30 (s, 2H).
**2-((3-Aminophenyl)thio)acetohydrazide, 9b.** White solid. Yield: 85%. M.p. 101-103 °C. ^{1}H-NMR (δ, ppm): 9,20 (s, 1H, exc.), 6,94 (t, 1H, J=7,96), 6,53 (d, 1H, J=1,94 Hz), 6,47 (d, 1H, J=8,32 Hz), 6,39 (dd, 1H, J=8.00 Hz, J=2,12), 5,13 (s, 2H, exc.), 4,20 (s, 2H, exc.), 3,502 (s, 2H).
**4-(((5-Phenyl-1*H*-1,2,4-triazol-3-yl)methyl)thio)aniline, 5a.** Filmy oil. Yield: 26%. ^{1}H-NMR (δ, ppm): 14.91 (s, 1H, exc.), 7.96 (d, 2H, J=8.35 Hz), 7.46 (m, 3H), 7.01 (d, 2H, J=8.21 Hz), 6.50 (d, 2H, J=8.41 Hz), 5.27 (s, 2H, exc.), 4.03 (s, 2H).
**4-(((5-(4-Chlorophenyl)-1*H*-1,2,4-triazol-3-yl)methyl)thio)aniline, 5b.** White solid. Cryst. solvent: EtOH. Yield: 72%. M.p. 168-170 °C. ^{1}H-NMR (δ, ppm): 14.03 (s, 1H, exc.), 7.96 (d, 2H, J=8.60 Hz), 7.54 (d, 2H, J=8.50 Hz), 7.07 (d, 2H, J=8.40 Hz), 6.47 (d, 2H, J=8.55 Hz), 5.30 (s, 2H, exc.), 4.04 (s, 2H).
**4-(((5-(4-Bromophenyl)-1*H*-1,2,4-triazol-3-yl)methyl)thio)aniline, 5c.** White solid. Cryst. solvent: EtOH. Yield: 46%. M.p. 102-103 °C. ^{1}H-NMR (δ, ppm): 13,91 (s, 1H, exc.), 7,90 (d, 2H, J=8.55 Hz), 7,67 (d, 2H, J=8.73 Hz), 7,06 (d, 2H, J=8.32 Hz), 6,48 (d, 2H, J=8.77 Hz), 5,29 (s, 2H, exc.), 4,04 (s, 2H).

**3-(((5-(4-Chlorophenyl)-1*H*-1,2,4-triazol-3-yl)methyl)thio)aniline, 5d.** White solid. Cryst. solvent: EtOH. Yield: 72%. M.p. 168-170 °C. ^{1}H-NMR (δ, ppm): 14,01 (s, 1H, exc.), 7,96 (d, 2H, J=8.34 Hz), 7,69 (d, 2H, J=8.56 Hz), 7,07 (d, 2H, J=8.36 Hz), 6,48 (d, 2H, J=7.95 Hz), 5,30 (s, 2H, exc.), 4,04 (s, 2H).

Table 3-SI. Physical and Spectral Data of 1,2,4-Triazole Inhibitors 6a-i.

**1-Phenyl-3-(4-(((3-phenyl-1*H*-1,2,4-triazol-3-yl)methyl)thio)phenyl)urea, 6a.** White solid. M.p. 205-206 °C. Cryst. solvent: MeOH. Yield: 24%. ^{1}H-NMR (δ, ppm): 14.10 (s, 1H, exc.), 8.80 (s, 1H, exc.), 8.75 (s, 1H, exc), 7.97 (dd, 2H, J=8.60 Hz, J=4.80 Hz), 7.54 (d, 2H, J=8.60 Hz), 7.45-7.41 (m, 4H), 7.34 (dd, 2H, J=8.70 Hz, J=4.70 Hz), 7.28 (t, 2H, J=7.70), 6.97 (t, 1H, J=7.30 Hz), 4.23 (s, 2H). ^{13}C-NMR (δ, ppm): 161.46, 154.80, 152.87, 140.05, 139.64, 132.10, 131.40, 130.64, 129.24, 126.31, 122.37, 119.19, 118.72.
**1-(4-(((5-(4-Chlorophenyl)-1*H*-1,2,4-triazol-3-yl)methyl)thio)phenyl)-3-phenylurea, 6b.** White solid. M.p. 188-189 °C. Cryst. solvent: MeOH. Yield: 24%. ^{1}H-NMR (δ, ppm): 8.80 (s, 1H, exc.), 8.75 (s, 1H, exc.), 7.97 (d, 2H, J=8.64 Hz), 7.54 (d, 2H, J=8.56 Hz), 7.44 (d, 2H, J=8.72 Hz), 7.42 (d, 2H, J=8.84 Hz), 7.34 (d, 2H, J=6.81 Hz), 7.28 (t, 2H, J=7.52 Hz), 6.97 (t, 1H, J=7.36 Hz). ^{13}C-NMR (δ, ppm): 156.50, 152.88, 140.06, 139.48, 134.29, 131.82, 129.39, 129.25, 129.08, 127.89, 126.97, 122.35, 119.18, 118.69.
**1-(4-(((5-(4-Bromophenyl)-1*H*-1,2,4-triazol-3-yl)methyl)thio)phenyl)-3-phenylurea, 6c.** White solid. M.p. 178-180 °C. Cryst. solvent: MeOH. Yield: 51%. ^{1}H-NMR (δ, ppm): 14.10 (s, 1H, exc.), 8.75 (s, 1H, exc.), 8.69 (s, 1H, exc.), 7.90 (d, 2H, J=8.52 Hz), 7.68 (d, 2H, J=8.48 Hz), 7.44 (d, 2H, J=8.64 Hz), 7.41 (d, 2H, J=8.98 Hz), 7.34 (d, 2H, J=8.69 Hz), 7.28 (t, 2H, J=8.32 Hz), 6.97 (t, 1H, J=7.32 Hz), 4.24 (s, 2H). ^{13}C-NMR (δ, ppm): 155.63, 143.82, 131.79, 129.42, 127.96, 126.54, 126.25, 123.67, 122.45, 122.18, 119.24, 118.73.

**1-(4-(((5-(4-Chlorophenyl)-1*H*-1,2,4-triazol-3-yl)methyl)thio)phenyl)-3-(4-(trifluoromethyl) phenyl)urea, 6d.** White solid. M.p. 201-202 °C. Cryst. solvent: MeOH. Yield: 39%. ^{1}H-NMR (δ, ppm): 14.11 (s, 1H, exc.), 8.60 (s, 1H, exc.), 8.31 (s, 1H, exc.), 7.97 (d, 2H, J=8.52 Hz), 7.55 (d, 2H, J=8.36 Hz), 7.39 (d, 2H, J=8.68 Hz), 7.32 (d, 2H, J=8.68 Hz), 7.24 (d, 2H, J=8.88 Hz), 6.69 (d, 2H, J=9.00 Hz), 4.31 (s, 2H). ^{13}C-NMR (δ, ppm): 152.63, 143.82, 131.79, 129.42, 127.96, 126.54, 126.36, 123.67, 122.40, 122.08, 119.44, 118.43, 45.91.
**1-(4-(((5-(4-Chlorophenyl)-1*H*-1,2,4-triazol-3-yl)methyl)thio)phenyl)-3-(4-(dimethylamino)phe nyl)urea, 6e.** White solid. M.p. 201-202 °C. Cryst. solvent: MeOH. Yield: 39%. ^{1}H-NMR (δ, ppm): 14.11 (s, 1H, exc.), 8.60 (s, 1H, exc.), 8.31 (s, 1H, exc.), 7.97 (d, 2H, J=8.52 Hz), 7.55 (d, 2H, J=8.36 Hz), 7.39 (d, 2H, J=8.68 Hz), 7.32 (d, 2H, J=8.68 Hz), 7.24 (d, 2H, J=8.88 Hz), 6.69 (d, 2H, J=9.00 Hz), 4.23 (s, 2H), 2.83 (s, 6H). ^{13}C-NMR (δ, ppm): 153.11, 147.01, 132.18, 131.54, 130.42, 129.75, 129.65, 129.34, 127.97, 120.71, 120.42, 119.06, 118.96, 113.68, 113.58, 41.15.
**1-(4-(((5-(4-Chlorophenyl)-1*H*-1,2,4-triazol-3-yl)methyl)thio)phenyl)-3-(2,5-dimethoxyphenyl) urea, 6f.** White solid. M.p. 178-180 °C. Cryst. solvent: MeOH. Yield: 25%. ^{1}H-NMR (δ, ppm): 14.13 (s, 1H, exc.), 9.42 (s, 1H, exc.), 8.26 (s, 1H, exc.), 7.97 (dd, 2H, J=8.60 Hz, J=2.40 Hz), 7.84 (d, 1H, J=3.00 Hz), 7.55 (d, 2H, J=8.40 Hz), 7.42 (d, 2H, J=8.70 Hz), 7.35 (d, 2H, J=8.70 Hz), 6.92 (d, 1H, J=8.80 Hz), 6.49 (dd, 1H, J=8.40 Hz, J=3.00 Hz), 4.23 (s, 2H), 3.82 (s, 3H), 3.69 (s, 3H). ^{13}C-NMR (δ, ppm): 153.74, 152.63, 131.96, 129.85, 129.62, 129.42, 129.14, 128.00, 126.48, 126.18, 123.33, 122.49, 121.91, 118.63, 117.86, 117.02, 116.42, 63.95, 56.79, 29.35.
**1-(4-(((5-(4-Bromophenyl)-1*H*-1,2,4-triazol-3-yl)methyl)thio)phenyl)-3-(4-(trifluoromethyl) phenyl)urea, 6g.** White solid. M.p. 241-242 °C. Cryst. solvent: MeOH. Yield: 45%. ^{1}H-NMR (δ, ppm): 14.12 (s, 1H, exc.), 9.13 (s, 1H, exc.), 8.88 (s, 1H, exc.), 7.91 (dd, 2H, J=8.56 Hz, J=4.76 Hz), 7.69 (d, 2H, J=4.68 Hz), 7.66 (d, 2H, J=6.80 Hz), 7.64 (d, 2H, 6.65 Hz), 7.43 (d, 2H, J=6.76

Hz), 7.36 (d, 2H, J=8.72 Hz), 4.25 (s, 2H). ^{13}C-NMR (δ, ppm): 152.86, 140.03, 132.59, 132.16, 131.70, 131.40, 130.07, 129.24, 128.41, 128.17, 122.38, 119.21, 118.73.
**1-(4-(((5-(4-Bromophenyl)-1*H*-1,2,4-triazol-3-yl)methyl)thio)phenyl)-3-(3,4-dimethoxyphenyl) urea, 6h.** White solid. M.p. 138-140 °C. Cryst. solvent: MeOH. Yield: 42%. ^{1}H-NMR (δ, ppm): 14.12 (s, 1H, exc.), 8.71 (s, 1H, exc.), 8.57 (s, 1H, exc.), 7.90 (d, 2H, J=8.52 Hz), 7.68 (d, 2H, J=8.36 Hz), 7.41 (d, 2H, J=8.72 Hz), 7.32 (d, 2H, 8.68 Hz), 7.19 (s, 1H), 6.86 (d, 2H, J=8.72 Hz), 4.23 (s, 2H), 3.74 (s, 3H), 3.71 (s, 3H). ^{13}C-NMR (δ, ppm): 158.74, 156.63, 131.96, 129.85, 129.62, 129.42, 129.14, 128.00, 126.48, 126.18, 123.33, 122.49, 121.91, 118.63, 117.86, 117.02, 116.42, 63.95, 56.79.
**1-(3-(((5-(4-Chlorophenyl)-1*H*-1,2,4-triazol-3-yl)methyl)thio)phenyl)-3-(4-fluorophenyl)urea, 6i.** White solid. M.p. 145-147 °C. Cryst. solvent: MeOH. Yield: 47%. ^{1}H-NMR (δ, ppm): 14.20 (s, 1H, exc.), 9.67 (s, 1H, exc.), 9.64 (s, 1H, exc.), 8.75 (d, 2H, J=8.68 Hz), 8.71 (s, 1H), 7.98 (d, 2H, J=8.52 Hz), 7.90 (d, 2H, J=8.48 Hz), 7.69 (d, 2H, 8.48 Hz), 7.57-7.47 (m, 3H), 4.35 (s, 2H). ^{13}C-NMR (δ, ppm): 158.97, 156.61, 154.54, 153.18, 136.49, 136.46, 135.97, 133.37, 127.81, 120.49, 120.41, 115.84, 115.62, 108.49, 52.12.
